# Enrichment of Dairy-Type Lamb Diet with Microencapsulated Omega-3 Fish Oil: Effects on Growth, Carcass Quality and Meat Fatty Acids

**DOI:** 10.3390/life13020275

**Published:** 2023-01-18

**Authors:** Davide De Marzo, Giancarlo Bozzo, Edmondo Ceci, Caterina Losacco, Michela Maria Dimuccio, Rifat Ullah Khan, Vito Laudadio, Vincenzo Tufarelli

**Affiliations:** 1 Department of Precision and Regenerative Medicine and Jonian Area (DiMePRe-J), Section of Veterinary Science and Animal Production, University of Bari ‘Aldo Moro’, Valenzano, 70010 Bari, Italy; 2 Department of Veterinary Medicine, University of Bari ‘Aldo Moro’, Valenzano, 70010 Bari, Italy; 3 Faculty of Animal Husbandry and Veterinary Sciences, College of Veterinary Sciences, The University of Agriculture, Peshawar 25000, Pakistan

**Keywords:** lamb, feeding, microencapsulated fish oil, growth, meat

## Abstract

The hypothesis that adding omega-3 oil to feedlot pellets will improve the meat’s favourable n-3 PUFA composition was tested in this experiment. Therefore, we evaluated the productive traits and modification of the composition of n-3 PUFA of *Longissimus lumborum* (LL) muscle in growing lambs supplemented with microencapsulated omega-3 oil (MEOIL) in pelleted total mixed rations (TMR). Thirty six one month old Valle del Belice male lambs (14.04 ± 0.1 kg) were randomly distributed to one of the three dietary treatments (*n* = 12 lambs each) and provided the supplemented diets up to 14 weeks of age: 1. control (CON) pelleted TMR without omega-3 oil supplementation; 2. omega-3 oil fortified pelleted TMR at 1% (MEOIL1) supplementation; and 3- Omega-3 oil fortified pelleted TMR at 3% (MEOIL3) supplementation. Supplementing MEOIL at both levels in diet positively impacted (*p* < 0.05) body weight (BW) and feed efficiency. At the end of feeding period, most carcass quality traits did not vary significantly (*p* > 0.05) among groups, with the exception of carcass dressing and loin yield at both levels of MEOIL. The color and physical traits of LL muscle were affected by MEOIL supplementation (*p* < 0.05), with no significant change in chemical characteristics. Fatty acids composition of meat in term of linolenic, eicosapentaenoic acid (EPA) and docosahexaenoic acid (DHA) were significantly (*p* < 0.05) influenced by both levels of MEOIL. It was concluded that the tested microencapsulated omega-3 oil preparation may be included at 1% in lamb diet for increasing unsaturated fatty acids in meat without any detrimental effects on lamb productivity.

## 1. Introduction

Light lambs are usually preferred by consumers in the Mediterranean region [1] for their low profile of fatty acids (FA). Many factors affect lamb tissue FA composition, including breed, sex, body weight, environmental condition and feeding management [2,3]. Furthermore, some trans-FAs present in the milk and meat are manufactured in response to bacterial biohydrogenation in the rumen and are garnering attention since they are nutritionally beneficial FAs [4,5]. In order to comply with modern nutritional trends, consumers prefer a balanced and healthful diet, with an emphasis on improved meat quality. Due to its biological benefits, meat high in omega-3 polyunsaturated fatty acids (n-3 PUFA) is considered as an important food item. Dietary n-3 PUFAs consumption prevents decline in immune response, reduces the incidence of cardiac diseases and breast cancer, and promotes brain health and fetus development [6]. For these reasons, studies on n-3 PUFAs are under way. Because the conversion of α-linolenic acid (α-LNA) into eicosapentaenoic acid (EPA) and docosahexaenoic acid (DHA) in humans is about 5–10% of the total amount [7], their daily dietary intake is considered essential. 

By lowering saturated fatty acid (SFA) and raising mono- and polyunsaturated fatty acid (MUFAs and PUFAs, respectively) concentrations in diet, it is possible to enhance the quality of lamb meat. This can lower the chances of plaque formation in the arteries of frequent meat consumers [8,9]. Furthermore, it has been shown that the PUFA content of lamb diets can be increased by providing PUFA-rich plant or animal origin oils [10]. However, few research trials have been done on the impact of feed additive encapsulated oil on the physical growth and carcass quality of lambs [11] and very little information is available on suitable supplemental doses of fish oil in lambs. However, a few studies have been conducted on the effects of encapsulated oil supplementation on growth and carcass traits [11]. The amount and type of fat supplied with diet, in particular, can alter unsaturated fatty acid (UFAs) and FA profiles in the rumen of ruminants [12]. Vegetable oils (from soybean, sunflower and canola) are commonly integrated into the diet of lambs [13], but few research reports are available on the FA profile of meat of lambs fed with fish oil. 

As a result, we hypothesized that including a microencapsulated fish oil preparation in the lamb diet would improve meat quality and beneficially modulate its FA profile, resulting in a healthier product for consumers. The aim of this study was to evaluate the effects of microencapsulated oil preparation inclusion on lamb growth, carcass traits, physicochemical characteristics and FA profile of lamb meat.

## 2. Materials and Methods

### 2.1. Dietary Treatments and Growth Performance

Total mixed rations (TMR) were formulated according to the nutritional formulation and requirements of growing lambs [14] and the three dietary treatments were as follows: (1) control (CON) pelleted TMR without omega-3 oil supplementation; (2) omega-3 oil fortified pelleted TMR at 1% (MEOIL1) supplementation; and (3) omega-3 oil fortified pelleted TMR at 3% (MEOIL3) supplementation (Table 1). The oil supplement was a protected (microencapsulated) and micro-pearled form of fish oil (Nordos Fat^®^, produced by Trouw Nutrition Italia S.p.A., Bussolengo, Verona, Italy). The FAs composition of the product is reported in Table 2 [15]. Diets were formulated using standard feed analysis to incorporate about 149 g/kg CP and 11.1 MJ/kg metabolizable energy (DM basis) as suggested by NRC (2007). The TMR was milled to 25 mm and then reduced to steam pellets (8 mm diameter) and incorporated into the daily diet [16,17].

After weaning period (30 ± 2.0 days old), a total of 36 dairy-type Valle del Belice male lambs (14.4 ± 0.1 kg) were equally and randomly distributed into three groups (control and two experimental groups). Lambs were reared in individual pens (2.2 1.0 m) and fed a calculated amount of 500 g of their assigned TMR per day (as-fed basis) and then rising gradually to about 3.5% of live weight [14]. After a seven-day adaption phase, the trial on lambs was initiated until the animals reached 14 weeks. The TMRs were divided into two equal meals and fed to the lambs. To avoid too much intake of dry matter (DMI), which leads to intestinal issues and death, rationed feeding was adopted [8]. Water that was fit for drinking was plentiful. Lamb weight was monitored weekly at 07:00 h before feeding. Each lamb’s daily refusals were used to calculate the DMI. All lambs were inspected by a veterinarian during the feeding trial after receiving an anti-clostridial vaccination and treatment for internal parasites prior to the start of the study.

### 2.2. Carcass Sampling

After 16 h of fasting, all lambs (14 weeks old) were weighed and then slaughtered according to the established procedures [18]. Lambs were completely bled, peeled, and the internal organs were removed and weighed. The carcass was eviscerated and weighed after the removal of internal organs and tissues. The digestive tract was emptied and weighed. To calculate empty BW, the digestive contents were subtracted from the slaughter weight. Immediately, the hot carcasses were weighed and stored at 4 °C for 24 h. Dressing yield was determined by dividing fasting weight by cold carcass weight [19]. The cold carcass was weighed before sawing the backbone vertically into two symmetrical halves. The right side of the carcasses were further divided into wholesale cuts (shoulder, neck, loin, leg, ribs, brisket) and weight of each cut was recorded separately. Bone, lean and dissectible cuts were removed and weighed.

### 2.3. Meat Traits 

Meat pH of *Longissimus lumborum* (LL) was recorded immediately after slaughter and repeated after 24 h (designated as pH_0_ and pH_24_, respectively) at 4 °C using a pH-meter. On LL muscle, meat tenderness and color were evaluated. The color of meat was evaluated in terms of brightness L*, redness a* and yellowness b* using a spectrophotometer [20]. The degree of meat tenderness was tested using an Instron 1140 apparatus (Instron, High Wycombe, UK) on both raw and cooked samples, through Warner-Bratzler shear (WBS) force. The cut sample was cylindrical in shape and had a 1.27-cm-diameter cut that was perpendicular to the path of the muscle fibers. Meat hardness was calculated using the force deformation curve that was found. Slices of a muscle that were 5 cm thick were taken and weighed both before and after the meat was cooked to a temperature of 75 °C inside a vented oven. In order to ascertain the nutritional makeup of meat, sub-samples of meat muscle were examined for lipids, protein, ash and moisture [21,22]. As previously stated by De Marzo et al. [8], 5 g of meat sample was freeze-dried, then crushed before the FA composition could be determined. The FAs were counted as a percentage of total FAs.

### 2.4. Statistical Analysis

The general linear model procedure was used to process data under completely randomized design using ANOVA [23]. Tukey’s test was employed to assess treatment mean differences for significant dietary effects. Unless otherwise noted, a *p* < 0.05 was used to determine significance difference.

## 3. Results 

### 3.1. Growth Performance

Supplementing the tested levels of MEOIL in the current experiment resulted in improved growth performance. It was observed that the final BW was significantly (*p* = 0.027) increased in lamb fed 3% of MEOIL compared to the other groups; moreover, compared to the control, lambs fed both MEOIL levels resulted in improved (*p* = 0.029) FCR (Table 3).

### 3.2. Carcass Quality and Meat Traits

The slaughtering data, carcass measurements and composition and meat cuts are reported in Table 4. At 14 weeks of age, empty BW was significantly (*p* = 0.034) increased in lamb group fed MEOIL3 compared to the other groups, whereas carcass dressing percentages (hot and cold) were improved (*p* = 0.041 and *p* = 0.046, respectively) in both supplemented groups in comparison to the control diet. Furthermore, supplementing MEOIL at both levels affected positively the loin cut yield (*p* = 0.043). Carcass measurements and composition were not affected by oil inclusion in the diet. The dietary treatments significantly influenced the lamb meat color (Table 5) in terms of lightness (L*) when fed both oil levels compared to the control (*p* < 0.032); the same was observed for WBS in cooked meat and for meat cooking loss of lambs fed MEOIL at both levels (Table 5). Chemically, the composition of meat was not influenced by dietary treatments. 

### 3.3. Lamb Meat Fatty Acids

The impact of fish oil supplementation on the FA composition of meat is reported in Table 6. Supplementing MEOIL at both levels led to significantly lower content of myristic acid (*p* = 0.023) and oleic acid (*p* = 0.036) compared to unsupplemented group. Conversely, heptadecanoic acid (*p* = 0.047), linolenic acid (*p* = 0.023), EPA (*p* = 0.039) and DHA (*p* = 0.021) was significantly higher in lambs fed both levels of oil compared to the control diet.

## 4. Discussion

### 4.1. Growth Performance and Carcass Characteristics

In the current study, final body weight was significantly higher in MEILO3; however, FCR was similar in the supplemented groups. Dietary changes had no negative impact on the health of lambs. Furthermore, no macroscopic lesions or pathological alterations were identified in the muscles and internal organs of lambs fed the various diets. Ruminant milk and meat have a very different FA composition from the offered nutrients because FAs produced from animal nutrients are processed in the rumen before absorption in the gut; this is why ruminants are referred to as “hetero-lipoid animals” [24]. The equilibrium between FA intake, synthesis, desaturation and esterification essentially determines the FA profile in animal products. The absorbed FAs and subsequent tissue deposition in the duodenum are significantly influenced by rumen digestion [25]. The current findings are consistent with the results of Ferreira et al. [26] and Hernández-Garca et al. [27]. Previously, fish oil concentrations of 10 g/kg or less, physical growth and feed efficiency were unchanged [28,29], while at concentrations of greater than 30 g/kg feed consumption and weight gain decreased [28]. Marinova et al. [28] found that a diet supplemented with 10 g/kg fish oil and 20 g/kg sunflower oil decreased fat percentage in loin cut and increased this in the shoulder. In contrast, Annett et al. [29] found that 35 g/kg fish oil increased the leg’s subcutaneous fat in lambs. Furthermore, due to the fat’s detrimental effects on the rumen microbial population, higher concentrations of fish oil decreased feed intake and, subsequently, average daily gain in ruminants.

### 4.2. Carcass and Meat Quality 

In the current study, meat color (L*) was improved in the supplemented groups. Changes in meat color are usually associated with difference in fat contents in the meat [30]. Ponanmpalam et al. [31] suggested that several factors including PUFAs affect meat color and stability. In the current study, shear force was significantly improved in the supplemented groups. These findings are similar to the previous reports on different sources of PUFA [32,33]. 

It is generally recognized that meat quality and human health are significantly influenced by the FAs makeup of the meat [34]. Consequently, modification of FAs of meat in productive animals has taken place through feeding in recent years. The fat content of the investigated beef muscle samples did not alter significantly in the current investigation, regardless of diet and this conclusion is consistent with Jaworska et al. [35] in lambs fed 10 g/kg of fish oil in diet. On the other hand, Marinova et al. [28] examined the impact of fish oil in lamb diets as additive and found alterations in the distribution of fat in the carcass. Their research suggests that PUFA may have an impact on lamb meat quality and carcass fatness while, without influencing lamb performance or meat cutting, dietary fish oil raises the fatty acid profile of meat.

### 4.3. Meat Fatty Acids Composition 

The present study on fatty acid profile analysis of lamb groups found that oleic and palmitic levels were the highest of all fatty acids tested. In subcutaneous fat and muscles, oleic acids and palmitic are the most abundant fatty acids. Furthermore, the smaller dose of fish oil had an effect on long-chain n-3 fatty acids. The diet fortification with fish oil enhanced the content of EPA and DHA. Because PUFAs are beneficial for human health, their increased presence in lamb meat demonstrates the crucial influence of n-3 fatty acid inclusion on meat quality in the animals’ diet. The decrease in C18:1 n-9 of LL muscle in the current study could be the cause of higher C18:3 n-3 contents [36]. It was suggested by Bessa et al. [37] that increased PUFA contents in the diet may result in inhibition of enzyme activity required for desaturation of C18:0. Similar observations were reported by Jeronimo et al. [38] that linseed oil effectively improved C18:3 n-3 FA in the muscle tissues. This also indicates that part of the fish oil could escape biohydrogenation and be absorbed in the rumen. Cooking loss in this study was reduced in the supplemented groups compared to the control. In general, cooking loss occurs from 15 to 40%. Meat with lower cooking loss has better meat quality because of less nutrients loss during the cooking. From our results, it can be inferred that addition of encapsulated fish oil has protective effects on cooking loss in lamb meat. Long-chain docosapentaenoic acid was found in meat samples and was more abundant in meat from lambs fed fish oil. Popova et al. [39] discovered that, when lambs were fed 2.5% fish oil in their diet, the concentrations of C16:1 and C18:1 increased, more significantly in the subcutaneous fat over the Longissimus dorsi, while C18:0 decreased. As a consequence of the alterations in the individual fatty acids, the amount of the SFA decreased while the sum of the MUFA increased in the lambs’ fat depots. Although fish oil is an expensive by-product ingredient, its addition lowers the grain consumption and improves the feed efficiency, leading to better partial net revenue per animal. The outcomes were similar to those observed in feedlot feeds for crossbred lambs with 12 g/kg fish oil [27]. Cost will govern inclusion levels because there will be a greater need for fishmeal in the future in industries including aquaculture, livestock feed and the production of pharmaceuticals and cosmetics.

## 5. Conclusions

The findings corroborate the meat lipid profile by showing that the tested oil preparation can be added at 10 g/kg to the lamb diet without negatively influencing growth traits or meat quality. Consequently, our research supported the idea that adding fish oil to the diets of dairy-type lambs would produce meat with a better functional value. To fully confirm our findings, additional research on the ruminal fermentation pattern, feed digestibility and blood properties is required. 

## Figures and Tables

**Table 1:** Ingredient and chemical composition of diets fed to lambs.

Ingredients (% as-Fed Basis)	Diet		
	CON	MEOIL1	MEOIL3
Corn	27.90	27.90	25.80
Barley	18.50	18.50	18.00
Wheat bran	8.50	8.50	9.10
Soybean meal, 44% CP	8.50	8.50	8.50
Oat hay	7.50	7.50	7.50
Wheat middlings	7.00	7.00	7.50
Dehydrated beet pulp	5.00	5.00	4.50
Soybean hulls	5.00	5.00	4.50
Sunflower meal	4.00	4.00	4.50
Field bean	3.50	3.50	3.50
Calcium carbonate	2.00	2.00	2.00
Soybean oil	1.00	-	-
Microencapsulated oil	-	1.00	3.00
Dicalcium phosphate	0.50	0.50	0.50
Sodium chloride	0.25	0.25	0.25
Vitamin-mineral premix ^1^	0.25	0.25	0.25
Calcium sulphate	0.20	0.20	0.20
Yeast	0.20	0.20	0.20
Magnesium carbonate	0.10	0.10	0.10
Sodium bicarbonate	0.10	0.10	0.10
Chemical composition (%)			
Dry matter	89.97	89.65	89.41
Crude protein	14.95	14.88	14.85
Ether extract	3.50	3.54	4.95
Neutral detergent fiber	19.01	19.10	18.78
Acid detergent fiber	10.80	10.87	10.68
Ash	6.90	6.93	6.90
Metabolizable energy, MJ/kg DM	11.02	11.05	11.17

CON = control diet, MEOIL1 = diet containing 1% of microencapsulated oil (Nordos fat^®^), and MEOIL3 = diet containing 5% of microencapsulated oil (Nordos fat^®^). ^1^ Supplied per kg of diet: vitamin A, 13,500 IU; vitamin D3, 2700 IU; vitamin E, 13.5 mg; vitamin B1, 8.44 mg; vitamin B2, 5.06 mg; vitamin B6, 2.02 mg; D-pantothenic acid, 6.75 mg; niacin, 21.93 mg; vitamin B12, 0.01 mg; Co, 0.51 mg; Fe, 67.5 mg; I, 1.65 mg; Mn, 40.5 mg; Se, 0.07 mg; Zn, 101.3 mg.

**Table 2:** Fatty acids composition of the microencapsulated oil (Nordos fat^®^) included in lamb diet ^1^.

Fatty Acids		%
Total lipids		84.16
Lauric acid	C12:0	0.12
Myristic acid	C14:0	3.10
Palmitic acid	C16:0	16.12
Palmitoleic acid	C16:1	0.48
Stearic acid	C18:0	37.72
Oleic acid	C18:1	4.64
Linoleic acid	C18:2 n-6	2.45
Linolenic acid	C18:3 n-3	11.00
Eicosanoic acid	C20:0	1.07
Gadoleic acid	C20:1	0.21
Dihomo-γ-linolenic acid	C20:3 n-6	0.18
Arachidonic acid	C20:4 n-6	1.19
Eicosapentaenoic acid	C20:5 n-3	5.98
Docosapentaenoic acid	C22:5 n-3	2.48
Docosahexaenoic acid	C22:6 n-3	11.15
Not identified	-	2.11
Total fatty acids		100.00
Σ SFA		58.13
Σ MUFA		5.33
Σ n-6 PUFA		3.82
Σ n-3 PUFA		30.61

^1^ Husveth et al. (2003); SFA = saturated fatty acids; MUFA = monounsaturated fatty acids. PUFA = polyunsaturated fatty acids.

**Table 3:** Effect of diets on growth performance of lambs fed different levels of microencapsulated oil.

Item	Diet			SEM	*p*-Value
	CON	MEOIL1	MEOIL3		
Initial BW, kg	14.4	14.5	14.4	0.276	0.898
Final BW, kg	26.2 ^b^	26.7 ^b^	28.8 ^a^	1.723	0.027
Average daily BW gain, g/d	197	203	207	12.01	0.061
Average daily feed intake, g/d	1153	1160	1185	19.49	0.177
Feed conversion ratio, g DM/g gain	5.85 ^b^	5.71 ^a^	5.72 ^a^	0.198	0.029

CON = control diet, MEOIL1 = diet containing 1% of microencapsulated oil, MEOIL3 = diet containing 5% of microencapsulated oil. ^a,b^ Means within a row with no common superscript differ significantly (*p* < 0.05).

**Table 4:** Effect of dietary treatments on carcass traits of lambs fed different levels of microencapsulated oil.

Item	Diet				
	CON	MEOIL1	MEOIL3	SEM	*p*-Value
Slaughtering data					
Empty BW, kg	25.0 ^b^	25.6 ^b^	27.3 ^a^	0.269	0.034
Hot carcass dressing ^1^	57.2 ^b^	58.1 ^a^	58.5 ^a^	0.598	0.041
Cold carcass dressing ^1^	56.2 ^b^	57.3 ^a^	57.4 ^a^	0.422	0.046
Skin ^1^	11.21	10.84	11.02	0.101	0.098
Head ^1^	5.13	5.34	5.57	0.087	0.111
Offal ^1,2^	4.69	5.01	5.19	0.065	0.099
Carcass measurements, cm					
External carcass length	75.0	76.2	75.8	0.602	0.121
Internal carcass length	60.5	60.7	61.0	0.430	0.098
Leg length	31.2	31.5	31.7	0.401	0.215
Chest width	21.1	21.7	21.2	0.392	0.259
Meat cuts					
Right-half carcass, kg	5.24	5.79	6.12	0.067	0.098
Neck ^3^	10.49	9.78	10.15	0.125	0.087
Shoulder ^3^	17.74	18.85	18.45	0.177	0.061
Leg ^3^	31.26	31.77	31.86	0.298	0.101
Loin ^3^	8.01 ^b^	9.84 ^a^	9.11 ^a^	0.102	0.043
Brisket ^3^	12.05	12.44	12.50	0.163	0.091
Ribs ^3^	12.91	12.83	12.68	0.190	0.101
Carcass composition					
Lean ^4^	0.603	0.604	0.596	0.065	0.076
Bone ^4^	0.088	0.093	0.095	0.088	0.159
Dissectible fat ^4^	0.309	0.303	0.309	0.176	0.112

CON = control diet, MEOIL1 = diet containing 1% of microencapsulated oil, MEOIL3 = diet containing 5% of microencapsulated oil. ^1^ Values expressed as % of empty BW. ^2^ Liver + heart + kidney + lung + spleen. ^3^ Values expressed as % of right half-carcass. ^4^ Values expressed as proportion of pelvic limb. ^a,b^ Means within a row with no common superscript differ significantly (*p* < 0.05).

**Table 5:** Effect of dietary treatments on physical and chemical parameters of meat muscle (*Longissimus lumborum*) of lambs fed different levels of microencapsulated oil.

Item	Diet				
	CON	MEOIL1	MEOIL3	SEM	*p*-Value
pH_0_	6.71	6.73	6.75	0.044	0.188
pH_24_	5.70	5.69	5.67	0.040	0.095
L*	38.62 ^b^	40.65 ^a^	41.91 ^a^	1.012	0.032
a*	17.52	17.13	17.09	0.165	0.082
b*	7.31	7.27	7.12	0.335	0.095
WBS raw, kg/cm^2^	2.97	3.13	3.66	0.149	0.078
WBS cooked, kg/cm^2^	3.73 ^b^	2.69 ^a^	2.52 ^a^	0.078	0.025
Cooking loss, %	14.32 ^b^	13.77 ^a^	13.85 ^a^	0.587	0.028
Chemical composition, %					
Moisture	74.23	74.22	74.21	0.089	0.377
Protein	19.21	19.12	19.11	0.097	0.241
Lipids	5.29	5.41	5.44	0.038	0.057
Ash	1.27	1.25	1.24	0.045	0.198

CON = control diet, MEOIL1 = diet containing 1% of microencapsulated oil, MEOIL3 = diet containing 5% of microencapsulated oil. pH_0_ at slaughter; pH_24_ at 24 h post-mortem; WBS, Warner–Bratzler shear force. ^a,b^ Means within a row with no common superscript differ significantly (*p* < 0.05).

**Table 6:** Effect of dietary treatments on meat muscle (*Longissimus lumborum*) fatty acid composition (% total FA methyl esters) of lambs fed different levels of microencapsulated oil.

Item	Diet				
	CON	MEOIL1	MEOIL3	SEM	*p*-Value
C10:0 Capric	0.33	0.13	0.21	0.077	0.052
C12:0 Lauric	0.21	0.16	0.22	0.022	0.104
C14:0 Myristic	4.73 ^b^	2.55 ^a^	3.09 ^a^	0.917	0.023
C15:0 Pentadecylic	0.56	0.47	0.58	0.070	0.211
C16:0 Palmitic	23.04	23.10	23.07	0.833	0.095
C17:0 Heptadecanoic	1.67 ^b^	3.16 ^a^	3.00 ^a^	0.159	0.047
C18:0 Stearic	18.30	18.95	18.55	0.321	0.083
C20:0 Arachidic	0.16	0.21	0.20	0.011	0.065
C16:1 n-7 Palmitoleic	1.95	1.70	1.81	0.077	0.088
C17:1 Heptadecenoic	0.76	0.66	0.65	0.035	0.195
C18:1 n-9 Oleic	42.31 ^a^	41.97 ^b^	41.82 ^b^	0.612	0.036
C18:2 n-6 Linoleic	3.43	3.74	3.63	0.418	0.095
C18:3 n-3 α-Linolenic	1.39 ^b^	1.48 ^a^	1.52 ^a^	0.031	0.023
C20:1 Eicosenoic	0.17	0.18	0.16	0.012	0.216
C20:5 n-3 EPA	0.58 ^b^	0.67 ^a^	0.64 ^a^	0.045	0.039
C22:6 n-3 DHA	0.42 ^b^	0.87 ^a^	0.85 ^a^	0.060	0.021
Σ SFA	48.90	48.73	48.92	1.003	0.061
Σ UFA	51.10	51.27	51.08	1.122	0.075
SFA/UFA	0.96	0.95	0.96	0.045	0.096

CON = control diet, MEOIL1 = diet containing 1% of microencapsulated oil, MEOIL3 = diet containing 5% of microencapsulated oil. EPA = eicosapentaenoic acid; DHA = docosahexaenoic acid. SFA = saturated fatty acids; UFA = unsaturated fatty acids. ^a,b^ Means within a row with no common superscript differ significantly (*p* < 0.05).

## Data Availability

Not applicable.
